# Supplementary figures and images for: Estimating multiplicity of infection, haplotype frequencies, and linkage disequilibria from multi-allelic markers for molecular disease surveillance
Source: PLoS One. 2025 May 27;20(5):e0321723. doi: 10.1371/journal.pone.0321723 (PMC12111651; doi:10.1371/journal.pone.0321723)

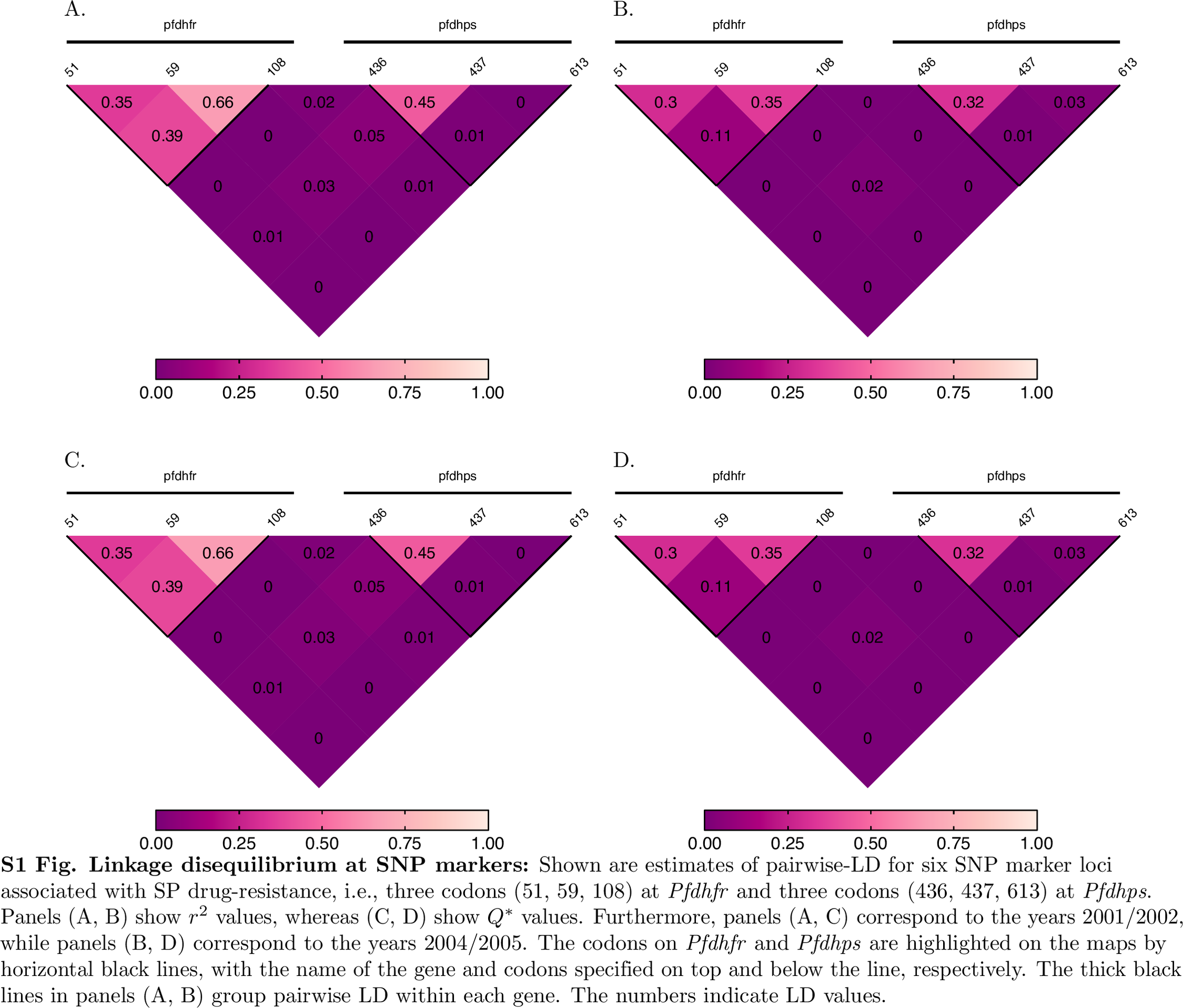

Supplement: S1 Fig — Shown are estimates of pairwise LD for six SNP marker loci associated with SP drug-resistance, i.e., three codons (51, 59, 108) at Pfdhfr and three codons (436, 437, 613) at Pfdhps. Panels (A, B) show r2 values, whereas (C, D) show Q* values. Furthermore, panels (A, C) correspond to the years 2001/2002, while panels (B, D) correspond to the years 2004/2005. The codons on Pfdhfr and Pfdhps are highlighted on the maps by horizontal black lines, with the name of the gene and codons are specified on top and below the line, respectively. The thick black lines in panels (A, B) group pairwise LD within each gene. The numbers indicate LD values. (TIF) [file pone.0321723.s001.tif]

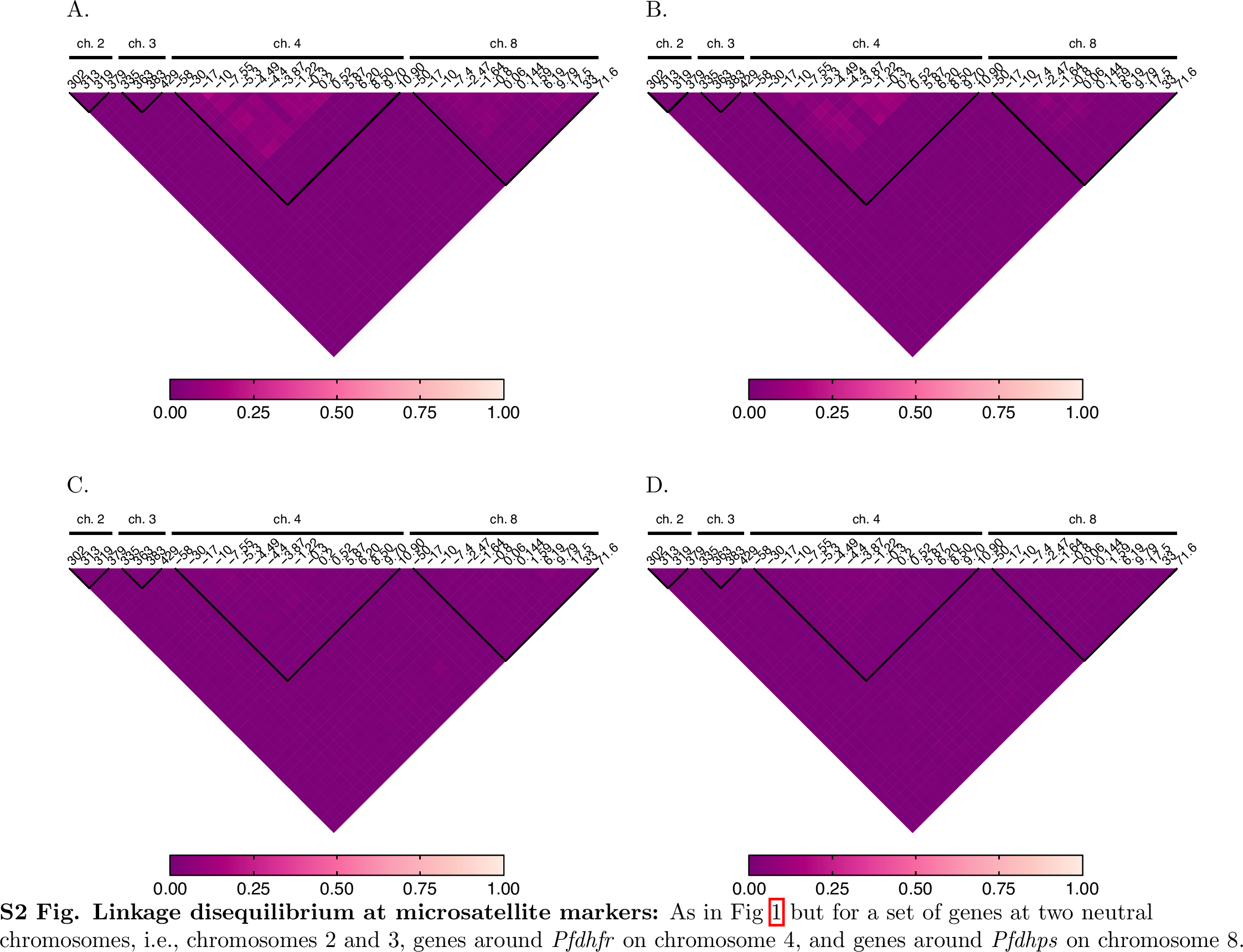

Supplement: S2 Fig — As in S1 Fig but for a set of genes at two neutral chromosomes, i.e., chromosomes 2 and 3, genes around Pfdhfr on chromosome 4, and genes around Pfdhps on chromosome 8. (TIF) [file pone.0321723.s002.tif]
