# Supplementary material for: Estimating multiplicity of infection, haplotype frequencies, and linkage disequilibria from multi-allelic markers for molecular disease surveillance
Source: PLoS One. 2025 May 27;20(5):e0321723. doi: 10.1371/journal.pone.0321723 (PMC12111651; doi:10.1371/journal.pone.0321723)
Supplement: S1 Mathematical appendix — (XLS) [file pone.0321723.s003.pdf]

Mathematical Appendix

Maximum-likelihood estimate

Here the EM-algorithm is derived. It consists of the E-step, which is derived first, and the M-step, which is derived afterward.

Expectation (E)-step

Each sample $\boldsymbol{x}^{(j)}$ in the dataset $\mathscr{X} = (\boldsymbol{x}^{(1)}, \ldots, \boldsymbol{x}^{(N)})$ results from a particular MOI vector $\boldsymbol{m}^{(j)}$, which is unobservable. We denote by $\mathscr{M} = (\boldsymbol{m}^{(1)}, \ldots, \boldsymbol{m}^{(N)})$ the MOI vectors associated with $\mathscr{X}$. Hence, the likelihood function $\ell_{\mathscr{X},\mathscr{M}}$ is defined as

$$\ell_{\mathscr{X},\mathscr{M}}(\boldsymbol{\theta}) := \prod_{j=1}^{N} P\big(\boldsymbol{x}^{(j)}, \boldsymbol{m}^{(j)} \big| \boldsymbol{\theta}\big). \tag{A.1}$$

The Q-function in step t is the expectation of the log-likelihood function of $\boldsymbol{\theta}$, given the observed and unobserved data $\mathscr{X}$ and $\mathscr{M}$ with respect to the conditional distribution of the unobserved data $\mathscr{M}$, given the observed data $\mathscr{X}$ and the parameter choice in the t-th step $\boldsymbol{\theta}_t$, i.e.,

$$\begin{aligned} Q(\boldsymbol{\theta} \,|\, \boldsymbol{\theta}_t) :=& \mathbb{E}_{\mathscr{M}|\mathscr{X},\boldsymbol{\theta}_t}[\log \ell_{\mathscr{X},\mathscr{M}}(\boldsymbol{\theta})] \\ =& \mathbb{E}_{\mathscr{M}|\mathscr{X},\boldsymbol{\theta}_t}\Big[\log \prod_{j=1}^{N} P\big(\boldsymbol{x}^{(j)}, \boldsymbol{m}^{(j)} \big| \boldsymbol{\theta}\big)\Big] \\ =& \mathbb{E}_{\mathscr{M}|\mathscr{X},\boldsymbol{\theta}_t}\Big[\sum_{j=1}^{N} \log P\big(\boldsymbol{x}^{(j)}, \boldsymbol{m}^{(j)} \big| \boldsymbol{\theta}\big)\Big] \\ =& \sum_{j=1}^{N} \mathbb{E}_{\mathscr{M}|\mathscr{X},\boldsymbol{\theta}_t}\big[\log P\big(\boldsymbol{x}^{(j)}, \boldsymbol{m}^{(j)} \big| \boldsymbol{\theta}\big)\big]. \end{aligned}$$

Note that

$$\begin{aligned} &\mathbb{E}_{\mathscr{M}|\mathscr{X},\boldsymbol{\theta}_t} \log \big[P\big(\boldsymbol{x}^{(j)}, \boldsymbol{m}^{(j)} \big| \boldsymbol{\theta}\big)\big] \\ &\qquad = \sum_{\boldsymbol{m}^{(1)},\ldots,\boldsymbol{m}^{(N)}} \log P\big(\boldsymbol{x}^{(j)}, \boldsymbol{m}^{(j)} \big| \boldsymbol{\theta}\big) P\big(\boldsymbol{m}^{(1)}, \ldots, \boldsymbol{m}^{(N)} \big| \boldsymbol{x}^{(1)}, \ldots, \boldsymbol{x}^{(N)}, \boldsymbol{\theta}_t\big) \\ &\qquad = \sum_{\boldsymbol{m}^{(1)},\ldots,\boldsymbol{m}^{(N)}} \log P\big(\boldsymbol{x}^{(j)}, \boldsymbol{m}^{(j)} \big| \boldsymbol{\theta}\big) \frac{P\Big(\big(\boldsymbol{x}^{(1)}, \boldsymbol{m}^{(1)}\big), \ldots, \big(\boldsymbol{x}^{(N)}, \boldsymbol{m}^{(N)}\big) \,|\, \boldsymbol{\theta}_t\Big)}{P\big(\boldsymbol{x}^{(1)}, \ldots, \boldsymbol{x}^{(N)} \big| \boldsymbol{\theta}_t\big)}. \end{aligned}$$

The samples in the observed and unobserved datasets $\mathscr{X}$ and $\mathscr{M}$ are independent. Hence,

$$\mathbb{E}_{\mathscr{M}|\mathscr{X},\boldsymbol{\theta}_t} \log \big[P\big(\boldsymbol{x}^{(j)}, \boldsymbol{m}^{(j)} \big| \boldsymbol{\theta}\big)\big] = \sum_{\boldsymbol{m}^{(1)},\ldots,\boldsymbol{m}^{(N)}} \log P\big(\boldsymbol{x}^{(j)}, \boldsymbol{m}^{(j)} \big| \boldsymbol{\theta}\big) \prod_{l=1}^{N} \frac{P\big(\boldsymbol{x}^{(l)}, \boldsymbol{m}^{(l)} \big| \boldsymbol{\theta}_t\big)}{P\big(\boldsymbol{x}^{(l)} \big| \boldsymbol{\theta}_t\big)}. \tag{A.2}$$

The factors in the above product are conditional probabilities, i.e.,

$$\frac{P\big(\boldsymbol{x}^{(l)}, \boldsymbol{m}^{(l)} \big| \boldsymbol{\theta}_t\big)}{P\big(\boldsymbol{x}^{(l)} \big| \boldsymbol{\theta}_t\big)} = \frac{P\big(\boldsymbol{x}^{(l)}, \boldsymbol{m}^{(l)}, \boldsymbol{\theta}_t\big) P\big(\boldsymbol{\theta}_t\big)}{P\big(\boldsymbol{x}^{(l)}, \boldsymbol{\theta}_t\big) P\big(\boldsymbol{\theta}_t\big)} = \frac{P\big(\boldsymbol{x}^{(l)}, \boldsymbol{m}^{(l)}, \boldsymbol{\theta}_t\big)}{P\big(\boldsymbol{x}^{(l)}, \boldsymbol{\theta}_t\big)} = P\big(\boldsymbol{m}^{(l)} \big| \boldsymbol{x}^{(l)}, \boldsymbol{\theta}_t\big).$$

Consequently, (A.2) simplifies to

$$\mathbb{E}_{\mathscr{M} | \mathscr{X}, \boldsymbol{\theta}_t} \big[\log P\big(\boldsymbol{x}^{(j)}, \boldsymbol{m}^{(j)} \big| \boldsymbol{\theta}\big) \big] = \sum_{\boldsymbol{m}^{(1)}, \ldots, \boldsymbol{m}^{(N)}} \big[\log P\big(\boldsymbol{x}^{(j)}, \boldsymbol{m}^{(j)} \big| \boldsymbol{\theta}\big) \big] \prod_{l=1}^{N} P\big(\boldsymbol{m}^{(l)} \big| \boldsymbol{x}^{(l)}, \boldsymbol{\theta}_t\big). \tag{A.3}$$

Furthermore,

$$\mathbb{E}_{\boldsymbol{m}^{(j)} | \boldsymbol{x}^{(j)}, \boldsymbol{\theta}_t} \big[\log P\big(\boldsymbol{x}^{(j)}, \boldsymbol{m}^{(j)}\big) \big] = \sum_{\boldsymbol{m}^{(j)}} \big[\log P\big(\boldsymbol{x}^{(j)}, \boldsymbol{m}^{(j)} \big| \boldsymbol{\theta}\big) \big] P\big(\boldsymbol{m}^{(j)} \big| \boldsymbol{x}^{(j)}, \boldsymbol{\theta}_t\big). \tag{A.4}$$

By rearranging the sum in (A.3) such that the innermost sum runs over all possible vectors $\boldsymbol{m}^{(j)}$ and using (A.4), one arrives at

$$\begin{aligned} &\mathbb{E}_{\mathscr{M} | \mathscr{X}, \boldsymbol{\theta}_t} \log \big[P\big(\boldsymbol{x}^{(j)}, \boldsymbol{m}^{(j)} \big| \boldsymbol{\theta}\big) \big] \\ &= \sum_{\substack{\boldsymbol{m}^{(1)}, \ldots, \boldsymbol{m}^{(j-1)}, \\ \boldsymbol{m}^{(j+1)}, \ldots, \boldsymbol{m}^{(N)}}} \mathbb{E}_{\boldsymbol{m}^{(j)} | \boldsymbol{x}^{(j)}, \boldsymbol{\theta}_t} \Big[\log P\big(\boldsymbol{x}^{(j)}, \boldsymbol{m}^{(j)} \big| \boldsymbol{\theta}\big) \Big] \prod_{\substack{l=1 \\ l \neq j}}^{N} P\big(\boldsymbol{m}^{(l)} \big| \boldsymbol{x}^{(l)}, \boldsymbol{\theta}_t\big) \\ &= \mathbb{E}_{\boldsymbol{m}^{(j)} | \boldsymbol{x}^{(j)}, \boldsymbol{\theta}_t} \Big[\log P\big(\boldsymbol{x}^{(j)}, \boldsymbol{m}^{(j)} \big| \boldsymbol{\theta}\big) \Big] \sum_{\substack{\boldsymbol{m}^{(1)}, \ldots, \boldsymbol{m}^{(j-1)}, \\ \boldsymbol{m}^{(j+1)}, \ldots, \boldsymbol{m}^{(N)}}} \prod_{\substack{l=1 \\ l \neq j}}^{N} P\big(\boldsymbol{m}^{(l)} \big| \boldsymbol{x}^{(l)}, \boldsymbol{\theta}_t\big). \end{aligned} \tag{A.5}$$

Since for all l

$$\sum_{\boldsymbol{m}^{(l)}} P\big(\boldsymbol{m}^{(l)} \big| \boldsymbol{x}^{(l)}, \boldsymbol{\theta}_t\big) = 1,$$

rearranging the sum in (A.5) gives

$$\sum_{\substack{\boldsymbol{m}^{(1)}, \ldots, \boldsymbol{m}^{(j-1)}, \\ \boldsymbol{m}^{(j+1)}, \ldots, \boldsymbol{m}^{(N)}}} \prod_{\substack{l=1 \\ l \neq j}}^{N} P\big(\boldsymbol{m}^{(l)} \big| \boldsymbol{x}^{(l)}, \boldsymbol{\theta}_t\big) = 1.$$

Therefore,

$$\mathbb{E}_{\mathscr{M} | \mathscr{X}, \boldsymbol{\theta}_t} \big[\log P\big(\boldsymbol{x}^{(j)}, \boldsymbol{m}^{(j)} \big| \boldsymbol{\theta}\big) \big] = \mathbb{E}_{\boldsymbol{m}^{(j)} | \boldsymbol{x}^{(j)}, \boldsymbol{\theta}_t} \big[\log P\big(\boldsymbol{x}^{(j)}, \boldsymbol{m}^{(j)} \big| \boldsymbol{\theta}\big) \big]. \tag{A.6}$$

Hence, the Q-function becomes

$$Q(\boldsymbol{\theta} \,|\, \boldsymbol{\theta}_t) = \sum_{j=1}^{N} \mathbb{E}_{\boldsymbol{m}^{(j)} | \boldsymbol{x}^{(j)}, \boldsymbol{\theta}_t} \big[\log P\big(\boldsymbol{x}^{(j)}, \boldsymbol{m}^{(j)} \big| \boldsymbol{\theta}\big) \big]. \tag{A.7}$$

Note that the same observation $\boldsymbol{x}$ might occur several times in the data. Let $n_{\boldsymbol{x}}$, be the number of times observation $\boldsymbol{x}$ occurs in the data $\mathscr{X}$. Using this notation, the Q-function can be rewritten as

$$Q(\boldsymbol{\theta} \,|\, \boldsymbol{\theta}_t) = \sum_{\boldsymbol{x} \in \mathscr{O}} n_{\boldsymbol{x}} \mathbb{E}_{\boldsymbol{m} | \boldsymbol{x}, \boldsymbol{\theta}_t} \big[\log P(\boldsymbol{x}, \boldsymbol{m} | \boldsymbol{\theta}) \big]. \tag{A.8a}$$

By defining

$$Q_{\boldsymbol{x}}(\boldsymbol{\theta}\,|\,\boldsymbol{\theta}_t) := \mathbb{E}_{\boldsymbol{m}|\boldsymbol{x},\boldsymbol{\theta}_t}\big[\log P(\boldsymbol{x},\boldsymbol{m}|\boldsymbol{\theta})\big], \tag{A.8b}$$

the Q-function becomes

$$Q(\boldsymbol{\theta}\,|\,\boldsymbol{\theta}_t) = \sum_{\boldsymbol{x}\in\mathscr{O}} n_{\boldsymbol{x}} Q_{\boldsymbol{x}}(\boldsymbol{\theta}\,|\,\boldsymbol{\theta}_t). \tag{A.8c}$$

To further simplify (A.8c), we first simplify the expression (A.8b). From (**??**) we obtain

$$Q_{\boldsymbol{x}}(\boldsymbol{\theta}\,|\,\boldsymbol{\theta}_t) = \mathbb{E}_{\boldsymbol{m}|\boldsymbol{x},\boldsymbol{\theta}_t}\big[\log P(\boldsymbol{x},\boldsymbol{m}|\boldsymbol{\theta})\big] = \sum_{\boldsymbol{m}\in M_{\boldsymbol{x}}^{(m)}} P(\boldsymbol{m}|\boldsymbol{x},\boldsymbol{\theta}_t)\log P(\boldsymbol{x},\boldsymbol{m}|\boldsymbol{\theta}), \tag{A.9a}$$

where the second equation is satisfied because $P(\boldsymbol{m}|\boldsymbol{x},\boldsymbol{\theta}_t) = 0$ if $\boldsymbol{m} \notin M_{\boldsymbol{x}}^{(m)}$ (defined in Eq. **??**). As shown above, $P(\boldsymbol{m}|\boldsymbol{x},\boldsymbol{\theta}_t) = \dfrac{P(\boldsymbol{x},\boldsymbol{m}|\boldsymbol{\theta}_t)}{P(\boldsymbol{x}|\boldsymbol{\theta}_t)}$. Combining this with (A.9a) yields

$$\begin{aligned} Q_{\boldsymbol{x}}(\boldsymbol{\theta}\,|\,\boldsymbol{\theta}_t) &= \sum_{\boldsymbol{m}\in M_{\boldsymbol{x}}^{(m)}} \frac{P(\boldsymbol{x},\boldsymbol{m}|\boldsymbol{\theta}_t)}{P(\boldsymbol{x}|\boldsymbol{\theta}_t)} \log P(\boldsymbol{x},\boldsymbol{m}|\boldsymbol{\theta}) \\ &= \frac{1}{P(\boldsymbol{x}|\boldsymbol{\theta}_t)} \sum_{\boldsymbol{m}\in M_{\boldsymbol{x}}^{(m)}} P(\boldsymbol{x},\boldsymbol{m}|\boldsymbol{\theta}_t)\log P(\boldsymbol{x},\boldsymbol{m}|\boldsymbol{\theta}). \end{aligned} \tag{A.9b}$$

Because $P(\boldsymbol{x},\boldsymbol{m}|\boldsymbol{\theta}_t) = P(\boldsymbol{x}|\boldsymbol{m},\boldsymbol{\theta}_t)P(\boldsymbol{m}|\boldsymbol{\theta}_t)$,

$$Q_{\boldsymbol{x}}(\boldsymbol{\theta}\,|\,\boldsymbol{\theta}_t) = \frac{1}{P(\boldsymbol{x}|\boldsymbol{\theta}_t)} \sum_{\boldsymbol{m}\in M_{\boldsymbol{x}}^{(m)}} P(\boldsymbol{x}|\boldsymbol{m},\boldsymbol{\theta}_t)P(\boldsymbol{m}|\boldsymbol{\theta}_t)\log\big[P(\boldsymbol{x}|\boldsymbol{m},\boldsymbol{\theta})P(\boldsymbol{m}|\boldsymbol{\theta})\big]. \tag{A.9c}$$

The sum in (A.9d) runs over all $\boldsymbol{m} \in M_{\boldsymbol{x}}^{(m)}$. For $\boldsymbol{m} \in M_{\boldsymbol{x}}^{(m)}$, clearly $P(\boldsymbol{x}|\boldsymbol{m},\boldsymbol{\theta}_t) = 1$ holds, such that

$$Q_{\boldsymbol{x}}(\boldsymbol{\theta}\,|\,\boldsymbol{\theta}_t) = \frac{1}{P(\boldsymbol{x}|\boldsymbol{\theta}_t)} \sum_{\boldsymbol{m}\in M_{\boldsymbol{x}}^{(m)}} P(\boldsymbol{m}|\boldsymbol{\theta}_t)\log P(\boldsymbol{m}|\boldsymbol{\theta}). \tag{A.9d}$$

Furthermore, the theorem of total probability yields

$$P(\boldsymbol{m}|\boldsymbol{\theta}_t) = \sum_{m=1}^{\infty} P(\boldsymbol{m}|m,\boldsymbol{\theta}_t)P(m|\boldsymbol{\theta}_t) \tag{A.9e}$$

and we define $\kappa_m^{(t)} := P(m|\boldsymbol{\theta}_t)$ (note that $\kappa_m = P(m|\boldsymbol{\theta})$). This and (**??**) allow to rewrite (A.9d) as

$$\begin{aligned} Q_{\boldsymbol{x}}(\boldsymbol{\theta}\,|\,\boldsymbol{\theta}_t) &= \frac{1}{P(\boldsymbol{x}|\boldsymbol{\theta}_t)} \sum_{m=1}^{\infty} \sum_{\boldsymbol{m}\in M_{\boldsymbol{x}}^{(m)}} P(\boldsymbol{m}|m,\boldsymbol{\theta}_t)P(m|\boldsymbol{\theta}_t)\log\big[P(\boldsymbol{m}|m,\boldsymbol{\theta})P(m|\boldsymbol{\theta})\big] \\ &= \frac{1}{P(\boldsymbol{x}|\boldsymbol{\theta}_t)} \sum_{m=1}^{\infty} \sum_{\boldsymbol{m}\in M_{\boldsymbol{x}}^{(m)}} \kappa_m^{(t)} \binom{m}{\boldsymbol{m}} \boldsymbol{p}_{\boldsymbol{h},t}^{\boldsymbol{m}} \log\left[\kappa_m \binom{m}{\boldsymbol{m}} \boldsymbol{p}^{\boldsymbol{m}}\right]. \end{aligned} \tag{A.9f}$$

The inclusion-exclusion principle as used in (??) leads to

$$Q_{\boldsymbol{x}}(\boldsymbol{\theta} \,|\, \boldsymbol{\theta}_t) = \frac{1}{P(\boldsymbol{x}|\boldsymbol{\theta}_t)} \sum_{m=1}^{\infty} \kappa_m^{(t)} \sum_{\boldsymbol{y} \in \mathscr{A}_{\boldsymbol{x}}} (-1)^{|\boldsymbol{x}|-|\boldsymbol{y}|} \sum_{\substack{\boldsymbol{m}:|\boldsymbol{m}|=m \\ m_{\boldsymbol{h}}=0 \text{ if } \boldsymbol{h} \notin A_{\boldsymbol{y}}}} \binom{m}{\boldsymbol{m}} \boldsymbol{p}_t^{\boldsymbol{m}} \log\left[\kappa_m \binom{m}{\boldsymbol{m}} \boldsymbol{p}^{\boldsymbol{m}}\right], \tag{A.10a}$$

where $|\boldsymbol{x}|$ and $|\boldsymbol{y}|$ are the cardinality of the sets $\boldsymbol{x}$ and $\boldsymbol{y}$, respectively. Therefore, multiplication with $P(\boldsymbol{x} \,|\, \boldsymbol{\theta}_t)$ gives

$$\begin{aligned}
&P(\boldsymbol{x} \,|\, \boldsymbol{\theta}_t) Q_{\boldsymbol{x}}(\boldsymbol{\theta} \,|\, \boldsymbol{\theta}_t) \\
&= \sum_{\boldsymbol{y} \in \mathscr{A}_{\boldsymbol{x}}} (-1)^{|\boldsymbol{x}|-|\boldsymbol{y}|} \sum_{m=1}^{\infty} \kappa_m^{(t)} \sum_{\substack{\boldsymbol{m}:|\boldsymbol{m}|=m \\ m_{\boldsymbol{h}}=0 \text{ if } \boldsymbol{h} \notin A_{\boldsymbol{y}}}} \binom{m}{\boldsymbol{m}} \boldsymbol{p}_t^{\boldsymbol{m}} \log\left[\kappa_m \binom{m}{\boldsymbol{m}} \boldsymbol{p}^{\boldsymbol{m}}\right] \\
&= \sum_{\boldsymbol{y} \in \mathscr{A}_{\boldsymbol{x}}} (-1)^{|\boldsymbol{x}|-|\boldsymbol{y}|} \sum_{m=1}^{\infty} \kappa_m^{(t)} \sum_{\substack{\boldsymbol{m}:|\boldsymbol{m}|=m \\ m_{\boldsymbol{h}}=0 \text{ if } \boldsymbol{h} \notin A_{\boldsymbol{y}}}} \binom{m}{\boldsymbol{m}} \boldsymbol{p}_t^{\boldsymbol{m}} \Big[\log \kappa_m + \log \binom{m}{\boldsymbol{m}} + \log \boldsymbol{p}^{\boldsymbol{m}}\Big] \\
&= \sum_{\boldsymbol{y} \in \mathscr{A}_{\boldsymbol{x}}} (-1)^{|\boldsymbol{x}|-|\boldsymbol{y}|} \sum_{m=1}^{\infty} \kappa_m^{(t)} \sum_{\substack{\boldsymbol{m}:|\boldsymbol{m}|=m \\ m_{\boldsymbol{h}}=0 \text{ if } \boldsymbol{h} \notin A_{\boldsymbol{y}}}} \binom{m}{\boldsymbol{m}} \boldsymbol{p}_t^{\boldsymbol{m}} \Big[\log\Big(\frac{1}{e^{\lambda}-1} \frac{\lambda^m}{m!}\Big) + \log \binom{m}{\boldsymbol{m}} + \log \boldsymbol{p}^{\boldsymbol{m}}\Big] \\
&= \sum_{\boldsymbol{y} \in \mathscr{A}_{\boldsymbol{x}}} (-1)^{|\boldsymbol{x}|-|\boldsymbol{y}|} \sum_{m=1}^{\infty} \kappa_m^{(t)} \sum_{\substack{\boldsymbol{m}:|\boldsymbol{m}|=m \\ m_{\boldsymbol{h}}=0 \text{ if } \boldsymbol{h} \notin A_{\boldsymbol{y}}}} \binom{m}{\boldsymbol{m}} \boldsymbol{p}_t^{\boldsymbol{m}} \Big[-\log(e^{\lambda}-1) + m \log \lambda + \log \boldsymbol{p}^{\boldsymbol{m}}\Big] + \tilde{A}_t,
\end{aligned}$$

where

$$\tilde{A}_t = \sum_{\boldsymbol{y} \in \mathscr{A}_{\boldsymbol{x}}} (-1)^{|\boldsymbol{x}|-|\boldsymbol{y}|} \sum_{m=1}^{\infty} \kappa_m^{(t)} \sum_{\substack{\boldsymbol{m}:|\boldsymbol{m}|=m \\ m_{\boldsymbol{h}}=0 \text{ if } \boldsymbol{h} \notin A_{\boldsymbol{y}}}} \binom{m}{\boldsymbol{m}} \boldsymbol{p}_t^{\boldsymbol{m}} \Big[-\log(m!) + \log \binom{m}{\boldsymbol{m}}\Big],$$

is independent of $\boldsymbol{\theta}$. Combining this with (A.10a) yields

$$\begin{aligned}
P(\boldsymbol{x} \,|\, \boldsymbol{\theta}_t) Q_{\boldsymbol{x}}(\boldsymbol{\theta} \,|\, \boldsymbol{\theta}_t) = &- \sum_{\boldsymbol{y} \in \mathscr{A}_{\boldsymbol{x}}} (-1)^{|\boldsymbol{x}|-|\boldsymbol{y}|} \sum_{m=1}^{\infty} \kappa_m^{(t)} \sum_{\substack{\boldsymbol{m}:|\boldsymbol{m}|=m \\ m_{\boldsymbol{h}}=0 \text{ if } \boldsymbol{h} \notin A_{\boldsymbol{y}}}} \binom{m}{\boldsymbol{m}} \boldsymbol{p}_t^{\boldsymbol{m}} \log(e^{\lambda}-1) \\
&+ \sum_{\boldsymbol{y} \in \mathscr{A}_{\boldsymbol{x}}} (-1)^{|\boldsymbol{x}|-|\boldsymbol{y}|} \sum_{m=1}^{\infty} m \kappa_m^{(t)} \sum_{\substack{\boldsymbol{m}:|\boldsymbol{m}|=m \\ m_{\boldsymbol{h}}=0 \text{ if } \boldsymbol{h} \notin A_{\boldsymbol{y}}}} \binom{m}{\boldsymbol{m}} \boldsymbol{p}_t^{\boldsymbol{m}} \log \lambda \\
&+ \sum_{\boldsymbol{y} \in \mathscr{A}_{\boldsymbol{x}}} (-1)^{|\boldsymbol{x}|-|\boldsymbol{y}|} \sum_{m=1}^{\infty} \kappa_m^{(t)} \sum_{\boldsymbol{h} \in \mathscr{H}} \log p_{\boldsymbol{h}} \sum_{\substack{\boldsymbol{m}:|\boldsymbol{m}|=m \\ m_{\boldsymbol{i}}=0 \text{ if } \boldsymbol{i} \notin A_{\boldsymbol{y}}}} m_{\boldsymbol{h}} \binom{m}{\boldsymbol{m}} \boldsymbol{p}_t^{\boldsymbol{m}} \\
&+ \tilde{A}_t.
\end{aligned} \tag{A.10b}$$

From (**??**) and the multinomial theorem we obtain

$$
\begin{aligned}
P(\boldsymbol{x}\,|\,\boldsymbol{\theta}_t)Q_{\boldsymbol{x}}(\boldsymbol{\theta}\,|\,\boldsymbol{\theta}_t) = &- P(\boldsymbol{x}\,|\,\boldsymbol{\theta}_t)\log\left(e^{\lambda}-1\right) \\
&+ \sum_{\boldsymbol{y}\in\mathscr{A}_{\boldsymbol{x}}}(-1)^{|\boldsymbol{x}|-|\boldsymbol{y}|}\sum_{m=1}^{\infty} m\kappa_m^{(t)}\Big(\sum_{\boldsymbol{i}\in A_{\boldsymbol{y}}} p_{\boldsymbol{i}}^{(t)}\Big)^m \log\lambda \\
&+ \sum_{\boldsymbol{y}\in\mathscr{A}_{\boldsymbol{x}}}(-1)^{|\boldsymbol{x}|-|\boldsymbol{y}|}\sum_{m=1}^{\infty}\kappa_m^{(t)}\sum_{\boldsymbol{h}\in\mathscr{H}}\log p_{\boldsymbol{h}}\sum_{\substack{\boldsymbol{m}:|\boldsymbol{m}|=m\\ m_{\boldsymbol{i}}=0 \text{ if } \boldsymbol{i}\notin A_{\boldsymbol{y}}}} m_{\boldsymbol{h}}\binom{m}{\boldsymbol{m}}\boldsymbol{p}_t^{\boldsymbol{m}} \\
&+ \tilde{A}_t.
\end{aligned} \tag{A.10c}
$$

To simplify the last row above, we introduce the notation $\boldsymbol{m}_{-\boldsymbol{h}}$, which is the MOI vector $\boldsymbol{m}$ with the component of haplotype $\boldsymbol{h}$ reduced by 1, i.e., $m_{\boldsymbol{h}}$ is replaced by $m_{\boldsymbol{h}}-1$. Therefore, by rearranging the binomial coefficient $\binom{m}{\boldsymbol{m}}$ we have

$$
\begin{aligned}
P(\boldsymbol{x}\,|\,\boldsymbol{\theta}_t)Q_{\boldsymbol{x}}(\boldsymbol{\theta}\,|\,\boldsymbol{\theta}_t) = &- P(\boldsymbol{x}\,|\,\boldsymbol{\theta}_t)\log\left(e^{\lambda}-1\right) \\
&+ \sum_{\boldsymbol{y}\in\mathscr{A}_{\boldsymbol{x}}}(-1)^{|\boldsymbol{x}|-|\boldsymbol{y}|}\sum_{m=1}^{\infty} m\kappa_m^{(t)}\Big(\sum_{\boldsymbol{i}\in A_{\boldsymbol{y}}} p_{\boldsymbol{i}}^{(t)}\Big)^m \log\lambda \\
&+ \sum_{\boldsymbol{y}\in\mathscr{A}_{\boldsymbol{x}}}(-1)^{|\boldsymbol{x}|-|\boldsymbol{y}|}\sum_{\boldsymbol{h}\in\mathscr{H}} p_{\boldsymbol{h}}^{(t)}\log p_{\boldsymbol{h}}\sum_{m=1}^{\infty}\kappa_m^{(t)} m\sum_{\substack{\boldsymbol{m}:|\boldsymbol{m}|=m\\ m_{\boldsymbol{i}}=0 \text{ if } \boldsymbol{i}\notin A_{\boldsymbol{y}}\\ m_{\boldsymbol{h}}>0}} \binom{m-1}{\boldsymbol{m}_{-\boldsymbol{h}}}\boldsymbol{p}_t^{\boldsymbol{m}_{-\boldsymbol{h}}} \\
&+ \tilde{A}_t.
\end{aligned}
$$

Rearrangement in the second line and using the multinomial theorem in the third line (together with the fact that $\boldsymbol{h}\in A_{\boldsymbol{y}}$ in the last sum) yields

$$
\begin{aligned}
P(\boldsymbol{x}\,|\,\boldsymbol{\theta}_t)Q_{\boldsymbol{x}}(\boldsymbol{\theta}\,|\,\boldsymbol{\theta}_t) = &- P(\boldsymbol{x}\,|\,\boldsymbol{\theta}_t)\log\left(e^{\lambda}-1\right) \\
&+ \sum_{\boldsymbol{y}\in\mathscr{A}_{\boldsymbol{x}}}(-1)^{|\boldsymbol{x}|-|\boldsymbol{y}|}\sum_{\boldsymbol{h}\in A_{\boldsymbol{y}}} p_{\boldsymbol{h}}^{(t)}\sum_{m=1}^{\infty} m\kappa_m^{(t)}\Big(\sum_{\boldsymbol{i}\in A_{\boldsymbol{y}}} p_{\boldsymbol{i}}^{(t)}\Big)^{m-1} \log\lambda \\
&+ \sum_{\boldsymbol{y}\in\mathscr{A}_{\boldsymbol{x}}}(-1)^{|\boldsymbol{x}|-|\boldsymbol{y}|}\sum_{\boldsymbol{h}\in\mathscr{H}} p_{\boldsymbol{h}}^{(t)}\log p_{\boldsymbol{h}}\sum_{m=1}^{\infty} m\kappa_m^{(t)}\Big(\sum_{\boldsymbol{i}\in A_{\boldsymbol{y}}} p_{\boldsymbol{i}}^{(t)}\Big)^{m-1} \\
&+ \tilde{A}_t.
\end{aligned} \tag{A.10d}
$$

Inside the sums in the second and third lines of the above equation we recognize derivatives. The fact that $\boldsymbol{h}\in A_{\boldsymbol{y}}$ and the definition of the PGF lead to

$$
\begin{aligned}
\sum_{m=1}^{\infty} m\kappa_m^{(t)}\Big(\sum_{\boldsymbol{i}\in A_{\boldsymbol{y}}} p_{\boldsymbol{i}}^{(t)}\Big)^{m-1} &= \sum_{m=1}^{\infty}\kappa_m^{(t)}\frac{\partial}{\partial p_{\boldsymbol{h}}^{(t)}}\Big(\sum_{\boldsymbol{i}\in A_{\boldsymbol{y}}} p_{\boldsymbol{i}}^{(t)}\Big)^{m} \\
&= \frac{\partial}{\partial p_{\boldsymbol{h}}^{(t)}}\sum_{m=1}^{\infty}\kappa_m^{(t)}\Big(\sum_{\boldsymbol{i}\in A_{\boldsymbol{y}}} p_{\boldsymbol{i}}^{(t)}\Big)^{m} \\
&= \frac{\partial}{\partial p_{\boldsymbol{h}}^{(t)}} G\Big(\sum_{\boldsymbol{i}\in A_{\boldsymbol{y}}} p_{\boldsymbol{i}}^{(t)}\Big).
\end{aligned} \tag{A.10e}
$$

Combination of (A.10e) and (A.10d) gives

$$\begin{aligned}P(\boldsymbol{x}\,|\,\boldsymbol{\theta}_t)Q_{\boldsymbol{x}}(\boldsymbol{\theta}\,|\,\boldsymbol{\theta}_t) =& - P(\boldsymbol{x}\,|\,\boldsymbol{\theta}_t)\log\left(e^{\lambda}-1\right)\\ &+\sum_{\boldsymbol{y}\in\mathscr{A}_{\boldsymbol{x}}}(-1)^{|\boldsymbol{x}|-|\boldsymbol{y}|}\sum_{\boldsymbol{h}\in A_{\boldsymbol{y}}} p_{\boldsymbol{h}}^{(t)}\frac{\partial}{\partial p_{\boldsymbol{h}}^{(t)}}G_t\Bigg(\sum_{\boldsymbol{i}\in A_{\boldsymbol{y}}} p_{\boldsymbol{i}}^{(t)}\Bigg)\log\lambda\\ &+\sum_{\boldsymbol{y}\in\mathscr{A}_{\boldsymbol{x}}}(-1)^{|\boldsymbol{x}|-|\boldsymbol{y}|}\sum_{\boldsymbol{h}\in\mathscr{H}} p_{\boldsymbol{h}}^{(t)}\log p_{\boldsymbol{h}}\frac{\partial}{\partial p_{\boldsymbol{h}}^{(t)}}G_t\Bigg(\sum_{\boldsymbol{i}\in A_{\boldsymbol{y}}} p_{\boldsymbol{i}}^{(t)}\Bigg)\\ &+\tilde{A}_t,\end{aligned} \tag{A.10f}$$

where the subscript t in G_t emphasizes the dependency of the probability generating function G on λ_t in iteration t. Therefore,

$$\begin{aligned}Q_{\boldsymbol{x}}(\boldsymbol{\theta}\,|\,\boldsymbol{\theta}_t) =& -\log\left(e^{\lambda}-1\right)\\ &+\frac{\sum\limits_{\boldsymbol{y}\in\mathscr{A}_{\boldsymbol{x}}}(-1)^{|\boldsymbol{x}|-|\boldsymbol{y}|}\sum\limits_{\boldsymbol{h}\in A_{\boldsymbol{y}}} p_{\boldsymbol{h}}^{(t)}\frac{\partial}{\partial p_{\boldsymbol{h}}^{(t)}}G_t\Big(\sum\limits_{\boldsymbol{i}\in A_{\boldsymbol{y}}} p_{\boldsymbol{i}}^{(t)}\Big)\log\lambda}{P(\boldsymbol{x}\,|\,\boldsymbol{\theta}_t)}\\ &+\frac{\sum\limits_{\boldsymbol{y}\in\mathscr{A}_{\boldsymbol{x}}}(-1)^{|\boldsymbol{x}|-|\boldsymbol{y}|}\sum\limits_{\boldsymbol{h}\in\mathscr{H}} p_{\boldsymbol{h}}^{(t)}\log p_{\boldsymbol{h}}\frac{\partial}{\partial p_{\boldsymbol{h}}^{(t)}}G_t\Big(\sum\limits_{\boldsymbol{i}\in A_{\boldsymbol{y}}} p_{\boldsymbol{i}}^{(t)}\Big)}{P(\boldsymbol{x}\,|\,\boldsymbol{\theta}_t)}\\ &+\tilde{\tilde{A}}_t,\end{aligned} \tag{A.11a}$$

where $\tilde{\tilde{A}}_t = \dfrac{\tilde{A}_t}{P(\boldsymbol{x}\,|\,\boldsymbol{\theta}_t)}$. Note that

$$\frac{\partial}{\partial p_{\boldsymbol{h}}^{(t)}}G_t\Bigg(\sum_{\boldsymbol{i}\in A_{\boldsymbol{y}}} p_{\boldsymbol{i}}^{(t)}\Bigg) = G_t'\Bigg(\sum_{\boldsymbol{i}\in A_{\boldsymbol{y}}} p_{\boldsymbol{i}}^{(t)}\Bigg) I_{A_{\boldsymbol{y}}}(\boldsymbol{h}), \tag{A.11b}$$

where

$$I_{A_{\boldsymbol{y}}}(\boldsymbol{h}) = \begin{cases}1 & \text{if } \boldsymbol{h}\in Ay,\\ 0 & \text{if } \boldsymbol{h}\notin Ay.\end{cases} \tag{A.11c}$$

Using this fact and the definition of $P(\boldsymbol{x}\,|\,\boldsymbol{\theta}_t)$ in (**??**) yields,

$$\begin{aligned}Q_{\boldsymbol{x}}(\boldsymbol{\theta}\,|\,\boldsymbol{\theta}_t) =& -\log(e^{\lambda}-1)\\ &+\frac{\sum\limits_{\boldsymbol{y}\in\mathscr{A}_{\boldsymbol{x}}}(-1)^{|\boldsymbol{x}|-|\boldsymbol{y}|}\sum\limits_{\boldsymbol{h}\in A_{\boldsymbol{y}}} p_{\boldsymbol{h}}^{(t)} G_t'\Big(\sum\limits_{\boldsymbol{i}\in A_{\boldsymbol{y}}} p_{\boldsymbol{i}}^{(t)}\Big)\log\lambda}{\sum\limits_{\boldsymbol{y}\in\mathscr{A}_{\boldsymbol{x}}}(-1)^{|\boldsymbol{x}|-|\boldsymbol{y}|}G_t\big(\sum\limits_{\boldsymbol{i}\in A_{\boldsymbol{y}}} p_{\boldsymbol{i}}^{(t)}\big)}\\ &+\frac{\sum\limits_{\boldsymbol{y}\in\mathscr{A}_{\boldsymbol{x}}}(-1)^{|\boldsymbol{x}|-|\boldsymbol{y}|}\sum\limits_{\boldsymbol{h}\in\mathscr{H}} p_{\boldsymbol{h}}^{(t)}\log p_{\boldsymbol{h}} G_t'\Big(\sum\limits_{\boldsymbol{i}\in A_{\boldsymbol{y}}} p_{\boldsymbol{i}}^{(t)}\Big) I_{A_{\boldsymbol{y}}}(\boldsymbol{h})}{\sum\limits_{\boldsymbol{y}\in\mathscr{A}_{\boldsymbol{x}}}(-1)^{|\boldsymbol{x}|-|\boldsymbol{y}|}G_t\Big(\sum\limits_{\boldsymbol{i}\in A_{\boldsymbol{y}}} p_{\boldsymbol{i}}^{(t)}\Big)} + \tilde{\tilde{A}}_t.\end{aligned} \tag{A.11d}$$

The Q-function in (A.8c) becomes

$$
\begin{aligned}
Q(\boldsymbol{\theta}\,|\,\boldsymbol{\theta}_t) = &- N \log\left(e^{\lambda}-1\right) \\
&+ \sum_{\boldsymbol{x}\in\mathscr{O}} n_{\boldsymbol{x}} \frac{\sum\limits_{\boldsymbol{y}\in\mathscr{A}_{\boldsymbol{x}}} (-1)^{|\boldsymbol{x}|-|\boldsymbol{y}|} \sum\limits_{\boldsymbol{h}\in A_{\boldsymbol{y}}} p_{\boldsymbol{h}}^{(t)} G_t'\left(\sum\limits_{\boldsymbol{i}\in A_{\boldsymbol{y}}} p_{\boldsymbol{i}}^{(t)}\right) \log\lambda}{\sum\limits_{\boldsymbol{y}\in\mathscr{A}_{\boldsymbol{x}}} (-1)^{|\boldsymbol{x}|-|\boldsymbol{y}|} G_t\left(\sum\limits_{\boldsymbol{i}\in A_{\boldsymbol{y}}} p_{\boldsymbol{i}}^{(t)}\right)} \\
&+ \sum_{\boldsymbol{x}\in\mathscr{O}} n_{\boldsymbol{x}} \frac{\sum\limits_{\boldsymbol{y}\in\mathscr{A}_{\boldsymbol{x}}} (-1)^{|\boldsymbol{x}|-|\boldsymbol{y}|} \sum\limits_{\boldsymbol{h}\in \mathscr{H}} p_{\boldsymbol{h}}^{(t)} \log p_{\boldsymbol{h}} G_t'\left(\sum\limits_{\boldsymbol{i}\in A_{\boldsymbol{y}}} p_{\boldsymbol{i}}^{(t)}\right) I_{A_{\boldsymbol{y}}}(\boldsymbol{h})}{\sum\limits_{\boldsymbol{y}\in\mathscr{A}_{\boldsymbol{x}}} (-1)^{|\boldsymbol{x}|-|\boldsymbol{y}|} G_t\left(\sum\limits_{\boldsymbol{i}\in A_{\boldsymbol{y}}} p_{\boldsymbol{i}}^{(t)}\right)} \\
&+ \tilde{\tilde{A}}_t.
\end{aligned} \tag{A.11e}
$$

Rearranging the sums in the third term yields

$$
\begin{aligned}
Q(\boldsymbol{\theta}\,|\,\boldsymbol{\theta}_t) = &- N \log\left(e^{\lambda}-1\right) \\
&+ \sum_{\boldsymbol{x}\in\mathscr{O}} n_{\boldsymbol{x}} \frac{\sum\limits_{\boldsymbol{y}\in\mathscr{A}_{\boldsymbol{x}}} (-1)^{|\boldsymbol{x}|-|\boldsymbol{y}|} \sum\limits_{\boldsymbol{h}\in A_{\boldsymbol{y}}} p_{\boldsymbol{h}}^{(t)} G_t'\left(\sum\limits_{\boldsymbol{i}\in A_{\boldsymbol{y}}} p_{\boldsymbol{i}}^{(t)}\right) \log\lambda}{\sum\limits_{\boldsymbol{y}\in\mathscr{A}_{\boldsymbol{x}}} (-1)^{|\boldsymbol{x}|-|\boldsymbol{y}|} G_t\left(\sum\limits_{\boldsymbol{i}\in A_{\boldsymbol{y}}} p_{\boldsymbol{i}}^{(t)}\right)} \\
&+ \sum_{\boldsymbol{h}\in\mathscr{H}} \frac{\sum\limits_{\boldsymbol{x}\in\mathscr{O}} n_{\boldsymbol{x}} \sum\limits_{\boldsymbol{y}\in\mathscr{A}_{\boldsymbol{x}}} (-1)^{|\boldsymbol{x}|-|\boldsymbol{y}|} p_{\boldsymbol{h}}^{(t)} \log p_{\boldsymbol{h}} G_t'\left(\sum\limits_{\boldsymbol{i}\in A_{\boldsymbol{y}}} p_{\boldsymbol{i}}^{(t)}\right) I_{A_{\boldsymbol{y}}}(\boldsymbol{h})}{\sum\limits_{\boldsymbol{y}\in\mathscr{A}_{\boldsymbol{x}}} (-1)^{|\boldsymbol{x}|-|\boldsymbol{y}|} G_t\left(\sum\limits_{\boldsymbol{i}\in A_{\boldsymbol{y}}} p_{\boldsymbol{i}}^{(t)}\right)} \\
&+ \tilde{\tilde{A}}_t.
\end{aligned} \tag{A.11f}
$$

Let

$$
B_t := \sum_{\boldsymbol{x}\in\mathscr{O}} n_{\boldsymbol{x}} \frac{\sum\limits_{\boldsymbol{y}\in\mathscr{A}_{\boldsymbol{x}}} (-1)^{|\boldsymbol{x}|-|\boldsymbol{y}|} \sum\limits_{\boldsymbol{h}\in A_{\boldsymbol{y}}} p_{\boldsymbol{h}}^{(t)} G_t'\left(\sum\limits_{\boldsymbol{i}\in A_{\boldsymbol{y}}} p_{\boldsymbol{i}}^{(t)}\right)}{\sum\limits_{\boldsymbol{y}\in\mathscr{A}_{\boldsymbol{x}}} (-1)^{|\boldsymbol{x}|-|\boldsymbol{y}|} G_t\left(\sum\limits_{\boldsymbol{i}\in A_{\boldsymbol{y}}} p_{\boldsymbol{i}}^{(t)}\right)}, \tag{A.12a}
$$

and

$$
C_{\boldsymbol{h}}^{(t)} := p_{\boldsymbol{h}}^{(t)} \sum_{\boldsymbol{x}\in\mathscr{O}} n_{\boldsymbol{x}} \frac{\sum\limits_{\boldsymbol{y}\in\mathscr{A}_{\boldsymbol{x}}} (-1)^{|\boldsymbol{x}|-|\boldsymbol{y}|} G_t'\left(\sum\limits_{\boldsymbol{i}\in A_{\boldsymbol{y}}} p_{\boldsymbol{i}}^{(t)}\right) I_{A_{\boldsymbol{y}}}(\boldsymbol{h})}{\sum\limits_{\boldsymbol{y}\in\mathscr{A}_{\boldsymbol{x}}} (-1)^{|\boldsymbol{x}|-|\boldsymbol{y}|} G_t\left(\sum\limits_{\boldsymbol{i}\in A_{\boldsymbol{y}}} p_{\boldsymbol{i}}^{(t)}\right)}, \tag{A.12b}
$$

such that the Q-function is rewritten as

$$
Q(\boldsymbol{\theta}\,|\,\boldsymbol{\theta}_t) = -N \log\left(e^{\lambda}-1\right) + B_t \log\lambda + \sum_{\boldsymbol{h}\in\mathscr{H}} C_{\boldsymbol{h}}^{(t)} \log p_{\boldsymbol{h}} + \tilde{\tilde{A}}_t. \tag{A.12c}
$$

Maximization (M)-step

The maximization of the Q-function is performed during the M-step of the EM-algorithm. The updated parameters $\boldsymbol{\theta}_{t+1}$ of the model parameters in iteration $t+1$ are obtained as

$$\boldsymbol{\theta}_{t+1} = \arg\max_{\boldsymbol{\theta}} Q(\boldsymbol{\theta} \,|\, \boldsymbol{\theta}_t).$$

We maximize the Q-function using the method of Lagrange-multipliers under the constraint $\sum\limits_{\boldsymbol{h} \in \mathscr{H}} p_{\boldsymbol{h}} = 1$. The Lagrangian function is

$$L(\boldsymbol{\theta} \,|\, \boldsymbol{\theta}_t) := Q(\boldsymbol{\theta} \,|\, \boldsymbol{\theta}_t) - \gamma\Big(\sum_{\boldsymbol{h} \in \mathscr{H}} p_{\boldsymbol{h}} - 1\Big),$$

where γ is the Lagrange multiplier. The parameters $\boldsymbol{\theta}_{t+1}$ at step $t+1$ of the EM-algorithm are found by solving

$$\nabla L(\boldsymbol{\theta} \,|\, \boldsymbol{\theta}_t) = \mathbf{0}.$$

The Lagrangian function is given by

$$L(\boldsymbol{\theta} \,|\, \boldsymbol{\theta}_t) = -N \log(e^{\lambda} - 1) + B_t \log \lambda + \sum_{\boldsymbol{h} \in \mathscr{H}} C_{\boldsymbol{h}}^{(t)} \log p_{\boldsymbol{h}} - \gamma\Big(\sum_{\boldsymbol{h} \in \mathscr{H}} p_{\boldsymbol{h}} - 1\Big) \tag{A.13}$$

$$+ \tilde{\tilde{A}}_t, \tag{A.14}$$

and its gradient by

$$\frac{\partial L(\boldsymbol{\theta} \,|\, \boldsymbol{\theta}_t)}{\partial \lambda} = -\frac{N e^{\lambda}}{e^{\lambda} - 1} + \frac{B_t}{\lambda}, \tag{A.15a}$$

$$\frac{\partial L(\boldsymbol{\theta} \,|\, \boldsymbol{\theta}_t)}{\partial p_{\boldsymbol{h}}} = C_{\boldsymbol{h}}^{(t)} \frac{1}{p_{\boldsymbol{h}}} - \gamma, \tag{A.15b}$$

and

$$\frac{\partial L(\boldsymbol{\theta} \,|\, \boldsymbol{\theta}_t)}{\partial \gamma} = -\sum_{\boldsymbol{h} \in \mathscr{H}} p_{\boldsymbol{h}} + 1. \tag{A.15c}$$

To obtain the parameter update $\boldsymbol{\theta}_{t+1}$, we equate the above components of the gradient to zero. Equating (A.15b) to 0 gives

$$p_{\boldsymbol{h}} = \frac{C_{\boldsymbol{h}}^{(t)}}{\gamma}.$$

Substituting this expression into (A.15c) and equating the resulting term to 0 yields

$$\gamma = \sum_{\boldsymbol{h} \in \mathscr{H}} C_{\boldsymbol{h}}^{(t)}.$$

Combining the last two equations gives the frequency of haplotype $\boldsymbol{h}$ at iteration $t+1$, namely

$$p_{\boldsymbol{h}}^{(t+1)} = \frac{C_{\boldsymbol{h}}^{(t)}}{\sum\limits_{\boldsymbol{h}\in\mathscr{H}} C_{\boldsymbol{h}}^{(t)}}. \tag{A.16}$$

The MOI parameter λ at step $t+1$ is obtained by equating (A.15a) to 0, i.e.,

$$-\frac{Ne^{\lambda}}{e^{\lambda}-1} + \frac{B_t}{\lambda} = 0.$$

This non-linear equation has no closed solution and needs to be solved numerically. We do so by the 1-dimensional Newton-Raphson method. Let the function f be defined as

$$f(\lambda) := \frac{\lambda e^{\lambda}}{e^{\lambda}-1} - \frac{B_t}{N}. \tag{A.17a}$$

The 1-dimensional Newton-Raphson method yields the recursion equation

$$\lambda_{\tau+1} = \lambda_{\tau} - \frac{f(\lambda_{\tau})}{f'(\lambda_{\tau})}, \tag{A.17b}$$

where

$$f'(\lambda) = \frac{(1+\lambda)(1-e^{-\lambda}) - \lambda}{(1-e^{-\lambda})^2}. \tag{A.17c}$$

Therefore, the recursion equation is given by

$$\lambda_{\tau+1} = \lambda_{\tau} - \frac{\lambda_{\tau} - \frac{B_t}{N}(1-e^{-\lambda_{\tau}})}{1+\lambda_{\tau} - \frac{\lambda_{\tau}}{1-e^{-\lambda_{\tau}}}}. \tag{A.17d}$$

The value of the MOI parameter λ_{t+1} at iteration $t+1$ of the EM-algorithm is the limit of the recursion (A.17d).

The EM-algorithm using a plugin estimate for the MOI parameter

If one prefers to use a plugin estimate for the MOI parameter λ, it is straightforward to adapt the EM-algorithm. In fact, most of the derivations remain valid and need just little adjustments. In particular, $\lambda_t = \lambda = \lambda_{\text{plugin}}$.

The Q-function changes into

$$Q(\boldsymbol{\theta}\,|\,\boldsymbol{\theta}_t) = \sum_{\boldsymbol{h}\in\mathscr{H}} C_{\boldsymbol{h}}^{(t)} \log p_{\boldsymbol{h}} + A_t^*, \tag{A.18}$$

where $\boldsymbol{\theta} = \boldsymbol{p}$, $\boldsymbol{\theta}_t = \boldsymbol{p}_t$,

$$C_{\boldsymbol{h}}^{(t)} = p_{\boldsymbol{h}}^{(t)} \sum_{\boldsymbol{x} \in \mathscr{O}} n_{\boldsymbol{x}} \frac{\sum\limits_{\boldsymbol{y} \in \mathscr{A}_{\boldsymbol{x}}} (-1)^{|\boldsymbol{x}|-|\boldsymbol{y}|} G'_{\lambda_{\text{plugin}}}\left(\sum\limits_{\boldsymbol{i} \in A_{\boldsymbol{y}}} p_{\boldsymbol{i}}^{(t)}\right) I_{A_{\boldsymbol{y}}}(\boldsymbol{h})}{\sum\limits_{\boldsymbol{y} \in \mathscr{A}_{\boldsymbol{x}}} (-1)^{|\boldsymbol{x}|-|\boldsymbol{y}|} G_{\lambda_{\text{plugin}}}\left(\sum\limits_{\boldsymbol{i} \in A_{\boldsymbol{y}}} p_{\boldsymbol{i}}^{(t)}\right)}, \tag{A.19}$$

and A_t^* is independent of the parameters $\boldsymbol{\theta}$.

The maximization step is adapted similarly and leads to the iteration

$$p_{\boldsymbol{h}}^{(t+1)} = \frac{C_{\boldsymbol{h}}^{(t)}}{\sum\limits_{\boldsymbol{h} \in \mathscr{H}} C_{\boldsymbol{h}}^{(t)}}. \tag{A.20}$$
