# Supplementary material for: Estimating multiplicity of infection, haplotype frequencies, and linkage disequilibria from multi-allelic markers for molecular disease surveillance
Source: PLoS One. 2025 May 27;20(5):e0321723. doi: 10.1371/journal.pone.0321723 (PMC12111651; doi:10.1371/journal.pone.0321723)
Supplement: S1 User Manual — (XLS) [file pone.0321723.s004.pdf]

User Manual

An implementation of the model described in the main manuscript is provided as an R script named "STRModel.R". The script contains functions for the estimation of haplotype frequencies, multiplicity of infection (MOI), and four linkage disequilibrium (LD) measures, i.e., D', R^2, Q^*, and the conditional measure (ALD) (denoted $W_{A|B}$ and $W_{B|A}$), from a pair of multi-allelic loci. Additionally, the code in the script "STR_MLE.R" serves as template for deriving the estimates from empirical data.

The scripts and some example datasets can be found on GitHub (https://github.com/Maths-against-Malaria/MultiAllelicBiLociModel).

Loading the R script

Suppose the main R script is stored in a directory "<PATH>/STRModel.R". First, the script has to be loaded in the R environment (e.g., R Studio, VS Code, or an R terminal) using the following code:

```
# Load external resources
    source("~/home/johndoe/Documents/src/STRModel.R")
```

Here, we assume that the <PATH> containing the R script is "/home/johndoe/Documents/".

Importing data

A dataset needs to be imported first (see below for the required format). Assume the dataset "example_dataset1.xlsx" (provided with the script) is downloaded and stored in the folder "Documents" whose path is given by "/home/johndoe/Documents/". The dataset can be imported using the R-package "openxlsx". However, the package is not present by default and might need to be installed using the following code:

```
# Install library "openxlsx"
install.packages("openxlsx")
```

The library is then loaded upon successful installation and the data imported using the code:

```
# Load library "openxlsx"
library(openxlsx)

# Import data
DATA <- read.xlsx("~/home/johndoe/Documents/example_dataset1.xlsx")
```

A more comprehensive documentation exists as a guide to use the package "openxlsx". Note that other packages can be used to import ".xlsx" files in R and represent a good alternative to the "openxlsx" package described above. Moreover, functions such as "read.csv" and "read.table" can be used to import data in the ".csv" and ".txt" formats, respectively.

Standard input format and data transformation

The methods are designed for data containing information from a pair of multi-allelic markers, e.g., microsatellites markers with n_1 and n_2 alleles, respectively. First, the desired data format is explained. Second, it is explained how custom data can be converted into this format.

For each record (corresponding to one sample) the data indicates the absence and presence of the alleles found at both molecular markers, in the following convention. At marker k, the absence and presence of the alleles corresponds to a 0-1 vector of length n_k ($k = 1, 2$). This corresponds to a binary number between 0 and $2^{n_k} - 1$, where the vector $\mathbf{0} = (0, \ldots, 0)$ corresponds to missing data, $(1, 0, \ldots, 0)$ indicates the presence of the first allele, and $(1, 1, \ldots, 1)$ indicates the presence of all alleles.

A dataset of sample size N is an array with N rows in which the entries are numbers from 0 to $2^{n_1} - 1$ and 0 to $2^{n_2} - 1$ at the first and second marker, respectively. As an example, the following dataset of sample size $N = 100$, with $n_1 = 2$ and $n_2 = 3$ alleles at the first and second marker has the correct format:

ID	Marker1	Marker2
ID1	3	7
ID2	2	5
ID3	1	2
⋮	⋮	⋮
ID99	0	0
ID100	0	3

Absence/presence of alleles at the first marker are encoded by numbers from 0 to $2^2 - 1 = 3$, and by numbers from 0 to $2^3 - 1 = 7$ at the second marker. For the second record (ID2) the number 2 at the first marker corresponds to the 0-1 vector (0,1), indicating the absence of the first and presence of the second allele, while the entry 5 at marker 2 corresponds to the 0-1-vector (1,0,1) that represents the presence of only the first and third allele. For the last record (ID100), entry 0 at marker 1 corresponds to the vector (0,0) indicating missing data, while entry 3 is equivalent to the 0-1 vector (1,1,0), indicating the presence of the first and second alleles.

This format is referred to as '***standard input format***'. The ID column in the dataset is optional and can be omitted.

If data is not present in the format outlined above, it needs to be transformed. This can be done easily if the data is already in the following 'more natural' format, for which each record (sample) is represented by multiple **consecutive** rows, which list the alleles found at the respective markers. The first column has to contain the sample ID.

Assume a dataset with four molecular markers and the STR alleles (corresponding to distinct sequence lengths): (i) 130, 133 at marker 1; (ii) 201, 207, 210 at marker 2; (iii) 89, 91, 94, 99 at marker 3; and (iv) 140, 145, 148 at marker 4. Assume the following structure:

ID	M1	M2	M3	M4
ID1	130	207	99	140
ID1	133	201		
ID1		210		
ID2	133	201	99	140
ID2	133	210	91	145
ID3	130	207	91	148
⋮	⋮	⋮	⋮	⋮
ID99			89	145
ID99			99	148
ID100		201	94	148
ID100		207		140

Consider sample ID1. The two alleles 130 and 133 correspond to the 0-1 vector (1,1) at marker 1, the three alleles 201, 207, and 210, to (1,1,1) at marker 2, the allele 99 to (1,0,0,0) at marker 3, and the allele 140 to (1,0,0) at marker 4.

The function "data_format(<DATA>, output.id=TRUE)" takes the data in this 'more natural' format and creates a list as an output, with the data transformed into the standard input format as the first element. The second element is a list, which contains a string vector with the alleles occurring at each marker. The third element contains a vector with the number of alleles found at each marker. The optional boolean argument "output.id" (default "output.id=TRUE") indicates whether the transformed dataset in the standard input format should contain the sample ID in the first column. The following code first imports the data /home/johndoe/Documents/example_dataset4.xlsx" into "DATA1", transforms it into the standard input format, and saves the output list as "Ex.data":

```
# Import data
DATA1 <- read.xlsx("~/home/johndoe/Documents/example_dataset4.xlsx")

# Transform data into the standard format
Ex.data <- data_format(DATA1)
```

```
##  $ Ex.data
##  $`data transformed`
##  ID    M1   M2   M3   M4
##  ID1    2    4    4    4
##  ID2    2    4    2    1
##  ID3    1    4    2    1
##  ...    ...  ...  ...  ...
##  ID98   2    4    2    1
##  ID99   3    4    4    5
##  ID100  1    5    4    4

##  $`Alleles per markers`
##  $`Alleles per markers`$M1
##  [1] "130" "133"

##  $`Alleles per markers`$M2
##  [1] "201" "207" "210"

##  $`Alleles per markers`$M3
##  [1] "89" "91" "94" "99"

##  $`Alleles per markers`$M4
##  [1] "140" "145" "148"

##  [[3]]
##  M1 M2 M3 M4
##   2  3  4  3
```

The first element of the above list is in the desired standard input format but contains more than 2 markers.

If data is given in a different format, the R-package **MLMOI** provides a flexible function to import the data into this format. This package also helps to detect data entry errors. To install, load, and access the documentation of the package, run:

```
# Install library "MLMOI"
  install.packages("MLMOI")

# Load library
  library("MLMOI")

# Consult documentation
  ?MLMOI
```

Haplotype frequencies and MOI estimates

Assume that the script "STRModel.R" and the data set <DATA> have been loaded (see above).

The function "mle(<DATA>, <n1n2>,...)" derives the maximum likelihood estimates (MLEs) of the 2-marker-haplotype frequencies and the MOI parameter. Input arguments are the dataset in ***standard input***

format (<DATA>), the vector containing the number of alleles at the first and second marker (<n1n2>), i.e., the genetic architecture, and several optional arguments.

The output of the function "mle(<DATA>, <n1n2>,...)" is a list with three elements: (i) the MOI parameter $\hat{\lambda}$; (ii) the non-zero haplotype frequencies $\hat{p}$; (iii) a matrix of all detected haplotypes with estimated non-vanishing frequency; and (iv) the number of samples without missing data used to obtain the MLEs. Alleles at marker k are denoted by the numbers $0, \ldots, n_k - 1$ $(k = 1, 2)$.

```
# Estimate MLEs
  mle(DATA, c(2,3))
```

```
## $lambda
##
## 0.1466995
##
## $p
##             00        01        02        10        11        12
## [1,] 0.1479238 0.1479238 0.2042203 0.1820436 0.1820436 0.1358449
##
## $haplotypes
##      [,1] [,2]
## [1,]    0    0
## [2,]    0    1
## [3,]    0    2
## [4,]    1    0
## [5,]    1    1
## [6,]    1    2
##
## $`Sample size`
## [1] 100
```

If <DATA> does not contain sample IDs in the first column, the argument "id = FALSE" must be specified (default "id = TRUE").

As an example, consider the list "Ex.data" created above. The data in ***standard input format*** is the first list element and contains information from 4 markers. To calculate estimates for markers 3 and 4, the respective columns (i.e., columns 4 and 5) of the dataset can be chosen. The following code provides the estimates:

```
### Selecting the data without the column for sample IDs:
data <- Ex.data[[1]][,c(4,5)]

### Selecting the number of alleles at markers 3 and 4:
GA <- Ex.data[[3]][c(3,4)]

### Estimating the MLEs
mle(data, GA, id = FALSE)
```

```
## $lambda
##
## 0.6678529
##
## $p
##              00         01         02        10         30         12
## [1,] 0.05745106 0.06900567 0.03513807 0.4171279 0.06464585 0.06144528
##              32         11         20         21        22
## [1,] 0.05098825 0.01544861 0.05202984 0.02273323 0.1539863
```

```
##
## $haplotypes
##       [,1] [,2]
##  [1,]    0    0
##  [2,]    0    1
##  [3,]    0    2
##  [4,]    1    0
##  [5,]    3    0
##  [6,]    1    2
##  [7,]    3    2
##  [8,]    1    1
##  [9,]    2    0
## [10,]    2    1
## [11,]    2    2
##
## $`Sample size`
## [1] 97
```

Haplotype frequencies using a plug-in estimate for MOI

The function "mle(<DATA>,<n1n2>,...)" allows to derive the MLEs for haplotype frequencies based on a plug-in value for the MOI parameter (e.g., it has been independently estimated), i.e., the function provides the profile-likelihood estimates for haplotype frequencies given a fixed value for the MOI parameter. The argument "plugin=$\hat{\lambda}_{\text{plugin}}$" (default "plugin=NULL") specifies that $\hat{\lambda}_{\text{plugin}}$ should be used as plug-in value for the MOI parameter.

Consider the data "example_dataset1.xlsx" (with $n_1 = 2$ and $n_2 = 3$ alleles for markers 1 and 2, respectively), previously loaded and stored as DATA. Assuming the plug-in $\hat{\lambda}_{\text{plugin}} = 0.2$ for the MOI parameter, MLEs for the haplotype frequencies are obtained by running the code:

```
mle(DATA, c(2,3), plugin = 0.2)
```

```
## $lambda
##
## 0.2
##
## $p
##             00        01        02        10        11        12
## [1,] 0.1478403 0.1478403 0.2044126 0.1820468 0.1820468 0.1358131
##
## $haplotypes
##      [,1] [,2]
## [1,]    0    0
## [2,]    0    1
## [3,]    0    2
## [4,]    1    0
## [5,]    1    1
## [6,]    1    2
##
## $`Sample size`
## [1] 100
```

Bias-corrected estimates

Biased-corrected estimates can be obtained by setting the option "BC=TRUE" (default "BC = FALSE"). The default is a bootstrap bias correction (default "method='bootstrap' ") based on 10 000 bootstrap repeats (default "Bbias = 10 000"). Alternatively, a jackknife bias correction can be obtained by setting the option "method='jackknife' ". (The jackknife bias-correction ignores the optional argument "Bbias".)

The following code provides the bias-corrected MLEs for the dataset DATA based on 15 000 bootstrap repeats:

```
mle(DATA, c(2,3), BC = TRUE, Bbias = 15000)
```

```
## $lambda
##
## 0.1448664
##
## $p
##             00        01        02        10        11        12
## [1,] 0.1476457 0.1478205 0.2044729 0.1824764 0.1819097 0.1356747
##
## $haplotypes
##      [,1] [,2]
## [1,]    0    0
## [2,]    0    1
## [3,]    0    2
## [4,]    1    0
## [5,]    1    1
## [6,]    1    2
##
## $`Sample size`
## [1] 100
```

The bias-corrected MLEs with the 'jackknife' method and a plug-in value of the MOI parameter are obtained as follows:

```
mle(DATA, c(2,3), plugin = 0.2, BC = TRUE, method = "jackknife")
```

```
## $lambda
##
## 0.2
##
## $p
##             00        01        02        10        11        12
## [1,] 0.1478968 0.1476953 0.2045045 0.1821711 0.1819933 0.1357389
##
## $haplotypes
##      [,1] [,2]
## [1,]    0    0
## [2,]    0    1
## [3,]    0    2
## [4,]    1    0
## [5,]    1    1
## [6,]    1    2
##
## $`Sample size`
## [1] 100
```

Bootstrap confidence intervals

Equally-tailed $(1-\alpha)\times 100\%$ bootstrap-percentile confidence intervals (CIs) [1] are outputted alongside the estimates if the option "CI=TRUE" is specified (default "CI=FALSE"). The default are $B = 10\,000$ bootstrap repeats (default "B=10 000") and $\alpha = 0.05$ (default "alpha=0.05"). In this case, the MOI parameter estimate is a vector that contains the MLE, and the upper and lower confidence points, except a plug-in estimate is provided. The haplotype frequencies are provided as an array with 3 columns, where the first column provides the estimates, while the second and third yield the lower and upper confidence points, respectively.

To obtain the estimates of the MOI parameter and haplotype frequencies with their corresponding 95% CIs based on 15 000 bootstrap repeats, one has to run:

```
mle(DATA, c(2,3), CI = TRUE, B = 15000)
```

```
## $lambda
##                  2.5%      97.5%
## 0.14669947 0.04858829 0.27565340
##
## $p
##                    2.5%     97.5%
## 00 0.1479238 0.08471595 0.2191917
## 01 0.1479238 0.08067984 0.2219839
## 02 0.2042203 0.12645067 0.2850759
## 10 0.1820436 0.11044417 0.2576321
## 11 0.1820436 0.10990826 0.2604331
## 12 0.1358449 0.07137319 0.2074471
##
## $haplotypes
##      [,1] [,2]
## [1,]    0    0
## [2,]    0    1
## [3,]    0    2
## [4,]    1    0
## [5,]    1    1
## [6,]    1    2
##
## $`Sample size`
## [1] 100
```

The following code provides the estimates with 90% CIs based on 20 000 bootstrap repeats:

```
mle(DATA, c(2,3), CI = TRUE, B = 20000, alpha = 0.1)
```

```
## $lambda
##                    5%        95%
## 0.14669947 0.04933604 0.25060905
##
## $p
##                      5%       95%
## 00 0.1479238 0.09221254 0.2070232
## 01 0.1479238 0.09232429 0.2085694
## 02 0.2042203 0.13892626 0.2712269
## 10 0.1820436 0.12141267 0.2458922
## 11 0.1820436 0.12059606 0.2472573
## 12 0.1358449 0.08095101 0.1957825
##
## $haplotypes
```

```
##      [,1] [,2]
## [1,]    0    0
## [2,]    0    1
## [3,]    0    2
## [4,]    1    0
## [5,]    1    1
## [6,]    1    2
##
## $`Sample size`
## [1] 100
```

Linkage disequilibrium (LD) estimates

With the function "ld(<DATA>,...)" LD measures can be derived based. It outputs four LD measures, namely, D', r^2, Q^*, and the ALD measures $W_{A|B}$ and $W_{B|A}$. Internally, the function calls the function "mle(<DATA>,<n1n2>,...)", and allows retaining optional arguments. If the LD measures should be based on bias-corrected frequency estimates, the option "BC=TRUE" (default "BC = FALSE") has to be set. The default is a bootstrap bias correction (default "method='bootstrap' ") based on 10 000 bootstrap repeats (default "Bbias = 10 000"). Alternatively, a jackknife bias correction can be obtained by setting the option "method='jackknife' ". (The jackknife bias-correction ignores the optional argument "Bbias".) If a plug-in value should be used for the MOI parameter the option "plugin=$\hat{\lambda}_{\text{plugin}}$" has to be specified (default "plugin=NULL").

Also, $(1-\alpha)\times 100\%$ bootstrap percentile CIs can be obtained for the LD measures by setting the option "CI=TRUE" (default "CI=FALSE"). The default are $B = 10\,000$ bootstrap repeats (default "B=10 000") and $\alpha = 0.05$ (default "alpha=0.05").

LD measures for the previously loaded data, DATA, alongside 95% CIs based on $B = 15\,000$ bootstrap replicates are obtained by running the code:

```
ld(DATA, c(2,3), CI = TRUE)
```

```
##                       2.5%         97.5%
## D'         0.1366585  0.03311191   0.3384459
## r^2        0.01050607 0.0006702836 0.06949526
## Q*         0.01040208 0.0006640142 0.06714261
## W[A | B] 0.1024991  0.02588983   0.2636195
## W[B | A] 0.1447816  0.03673931   0.3732486
```

The following code provides the LD measures, based on a plug-in value $\hat{\lambda}_{\text{plugin}} = 0.2$ and bootstrap bias-corrected estimates based on $B = 500$ bootstrap repeats.

```
ld(DATA, c(2,3), plugin=0.2, CI = TRUE, BC = TRUE, Bbias = 500)
```

```
##                       2.5%         97.5%
## D'         0.1348244  0.03150679   0.340114
## r^2        0.01022618 0.0006291618 0.07006737
## Q*         0.0101296  0.0006200809 0.069024
## W[A | B] 0.1011246  0.02508309   0.2647024
## W[B | A] 0.1427041  0.03545448   0.3773453
```

Generating simulated data

Simulated data can be generated using the function "datasetgen(<P>,<lambda>,<N>,<arch>)". The required arguments are the haplotype frequency distribution $\boldsymbol{p}$ of length $n_1 n_2$, the MOI parameter λ, the sample size N, and a vector *arch* containing the number of alleles at the first and second locus, respectively.

For example, when considering $n_1 = 2$ and $n_2 = 3$ alleles at the first and second locus, respectively, haplotype frequency distribution $\boldsymbol{p} = (0.25, 0.15, 0.20, 0.10, 0.20, 0.10)$, and MOI parameter $\lambda = 0.2$, a dataset of sample size $N = 100$ is generated with the following code:

```
# Generate dataset
datasetgen(c(0.25,0.15,0.20,0.10,0.00,0.30), 0.2, 100, c(2,3))
```

The output (not shown here,) is a random $N \times 2$ matrix with entries corresponding to the data structure described above.

Regarding the frequency vector $\boldsymbol{p}$, the haplotypes have to be in a specific order. The function "hapl(*arch*)" obtains the required order. The function outputs a $n_1 n_2 \times 2$ matrix with entries 0 to $n_1 - 1$ at the first locus and 0 to $n_2 - 1$ at the second. Each row can be interpreted as the mix-radix representation (plus one) of the haplotype index in the base (n_1, n_2). For example, for $n_1 = 2$ and $n_2 = 3$ loci the code outputs the representation of all 6 possible haplotypes:

```
hapl(c(2,3))
```

```
##      [,1] [,2]
## [1,]    0    0
## [2,]    0    1
## [3,]    0    2
## [4,]    1    0
## [5,]    1    1
## [6,]    1    2
```

This indicates that haplotype (0,0) is represented by 1, haplotype (0,1) by 2, haplotype (0,2) by 3, haplotype (1,0) by 4, and so on. Hence, in the above code example $\boldsymbol{p} = (0.20, 0.15, 0.20, 0.10, 0.0, 0.30)$ indicates that haplotype (0,0) has frequency $p_1 = 0.2$, haplotype (0,1) has frequency $p_2 = 0.15$ haplotype (0,2) has frequency $p_3 = 0.2$ haplotype (1,0) has frequency $p_4 = 0.1$, and p_5, p_6 have frequency 0.00 and 0.30, respectively.

References

[1] Efron B, Tibshirani RJ. An Introduction to the Bootstrap. New York: Chapman and Hall/CRC; 1994.
