# Supplementary material for: Estimating multiplicity of infection, haplotype frequencies, and linkage disequilibria from multi-allelic markers for molecular disease surveillance
Source: PLoS One. 2025 May 27;20(5):e0321723. doi: 10.1371/journal.pone.0321723 (PMC12111651; doi:10.1371/journal.pone.0321723)
Supplement: Attachment — Submitted filename: PONE-D-23-29177_Review_.docx [file pone.0321723.s006.docx]

**Review of Manuscript PONE-D-23-29177**

**Title:** Estimating multiplicity of infection, haplotype frequencies, and linkage disequilibria from multi-allelic markers for molecular disease surveillance

**Overall Summary:** The manuscript presents a statistical framework for estimating haplotype frequencies and multiplicity of infection (MOI) from multi-allelic molecular markers, leveraging the maximum-likelihood method and the expectation-maximization (EM) algorithm. The study is well-motivated, addressing critical challenges in molecular disease surveillance, particularly in diseases like malaria. It demonstrates methodological rigor and is complemented by numerical simulations and a real-world application to sulfadoxine-pyrimethamine resistance in *Plasmodium falciparum* from Cameroon. The manuscript offers an innovative solution to a complex problem and makes a valuable contribution to the field.

**Technical Feedback:**

1. **Methodological Soundness:**
   - The use of the EM algorithm to estimate haplotype frequencies and MOI is well-justified, given the absence of a closed-form solution for maximum-likelihood estimates. The mathematical derivations are comprehensive and technically accurate.
   - The adaptation of the method to account for multi-allelic loci and ambiguous observations due to MOI is a strong innovation. This addresses a key gap in traditional population genetics tools, which often fail to handle polyploid-like behavior in malaria infections effectively.
2. **Simulation Study:**
   - The choice of parameters (e.g., allele numbers, sample sizes, MOI distributions) is appropriate and relevant for real-world malaria data. The extensive simulations across balanced and unbalanced haplotype frequency distributions provide robust evidence of the method’s accuracy and precision.
   - However, the manuscript could benefit from additional discussion on computational efficiency. While the EM algorithm is known for stability, its convergence in high-dimensional parameter spaces can be slow. Quantitative metrics on runtime performance would enhance the utility of the work.
3. **Real-World Application:**
   - The application to sulfadoxine-pyrimethamine resistance in *P. falciparum* is well-aligned with the study’s objectives and demonstrates the method’s relevance. The LD maps effectively capture evolutionary dynamics and drug pressure effects.
   - The discussion around high LD in regions flanking *Pfdhfr* and *Pfdhps* is compelling. However, the manuscript could explore potential confounding factors in the observed LD patterns, such as sampling bias or population substructure.
4. **Linkage Disequilibrium Measures:**
   - The use of multiple LD measures (D′, r², Q∗, and asymmetric conditional LD) is a strength, as it offers diverse perspectives on genetic associations. The manuscript could further elaborate on the comparative advantages and limitations of these measures, especially for multi-allelic loci.

**Clarity:**

1. **Abstract and Introduction:**
   - The abstract succinctly summarizes the study’s objectives, methods, and key findings. However, it could briefly mention the practical implications of the findings, particularly their relevance for disease control policies.
   - The introduction effectively sets the stage by highlighting the challenges of MOI and LD estimation in malaria. The transition from problem statement to proposed solution is clear and logical.
2. **Methodology:**
   - The mathematical details in the methods section are thorough but may be dense for readers unfamiliar with advanced statistical modeling. Consider adding a conceptual overview or visual aids (e.g., flowcharts) to complement the equations.
   - Definitions of key terms (e.g., MOI, haplotype frequencies) are clear, but the distinction between “super-infections” and “co-infections” could be expanded for clarity.
3. **Results and Figures:**
   - Figures are well-designed and enhance the manuscript’s readability. The LD maps (Figures 2 and 3) are particularly informative. Adding annotations to highlight key observations could further improve their interpretability.
   - The text accompanying the results is concise, but some sections (e.g., bias and variance analysis) would benefit from additional context to aid readers in interpreting the findings.
4. **Discussion and Conclusion:**
   - The discussion is well-structured, linking the findings to broader implications for molecular disease surveillance. It appropriately acknowledges limitations, such as the impact of small sample sizes and unbalanced haplotype frequencies.
   - The conclusion effectively reiterates the study’s contributions but could offer more specific recommendations for future research or practical applications.

**Novelty:**

1. The manuscript addresses a significant gap in the estimation of MOI and haplotype frequencies from multi-allelic data, which is critical for molecular disease surveillance.
2. The integration of multi-allelic LD measures and the application to real-world malaria data highlight the framework’s versatility and practical value.
3. The implementation of the method as an open-source R script enhances its accessibility and potential for adoption by the research community.

**Suggestions for Improvement:**

1. **Expand on Practical Applications:** Provide examples of how the proposed method can inform public health strategies, such as monitoring drug resistance or optimizing intervention programs.
2. **Enhance Accessibility:** Include a flowchart summarizing the methodological steps and a brief guide for non-specialists to understand the key concepts.
3. **Address Computational Aspects:** Discuss runtime performance and scalability for larger datasets, as this is a critical consideration for field applications.
4. **Supplementary Materials:** While the manuscript mentions a user manual, including a brief tutorial in the main text on using the R script would make the method more accessible.
